# 

**Published:** 1893-02-04

**Authors:** 


					4,1893. THE HOSPITAL,
**?*.?. . CONTENTS.
ifeSfiWW. of Disease
291
I.?The Bombay Pre'idency
292
^e<Ucal tri *??The Bombay Pre* idericy ?
^iTBTv^^torZ from the Earliest Times.
By E. T.
With.trom the Eai
tte<&T0N- M.A... M.B.Oxon.
XLI. ? Medirayal Military
Codeine
293
c?ateuv. 6 ? - ? ?
m, Theory and Research.-
-The Recognition of
TH(
1 Attu.1
-Naphtbalin as a Sedativa
294
as a Sedative
*. The pi_zrLe ?f, ?reat Writers towards Disease.
XX.-
The Pio? " oi U-reat Writers towards Disease. XX.?
w Xhe ureat w
?ver.Vtn^.n_e 2? Milan, 1630
295
SS&S582S
296
?A Masseuse's "Seoret"?Jack Tar's Cookerj*?
*? /t?spit?l s?V j JUaBsense a " Secret -
??tes bm iMurday in London - - ?
'?Jack Tar fl Cookery?
297
5?tea^~tnrdt
$?yal &wa
298
goyal
Benevolent College
Bene.
-Margarine Aaulteration
Sue
?*aPs aad ^eMSn"^"Marfi'arine ^?QU^era^on
306
The Hospital Clinic?
Rot al Infirmary, Edinburgh.
-Treatment of Pott s Fraotnre
Paddington Gbeen Childben's Hospital.
?The Treatment of
Hernia
299
St. Bartholomew's Hospital.-
The Treatment of Strangulated
Hernia
SOI
The Pbactitioner's bookshelf.-
- 1 The Diseases of Ohilaren:
Medical and Surgical
302
New Dbugs, Appliances, and Things Medical-
-The Newton-
Nixon Thermometer Oase
302
The Institutional Workshop-
Within thjs Hospitals -
-Bedford and its Medioal Institutions
303
HOSP TAL 1 CONSTRUCTION.-
-The Mildmay Medical Mission
Hospital, Austin Street, Shoreditcb
303
OOITSTEUOTIOH NOTE*
305
The Student of Mefticine.?Yaoancies?rasa Lasts.
EXTRA SUPPLEMENT.? THE NURSING MIRROR.
?ew8fr -EXTRA S TJ P P Ii E Jtt JS J
Hoapit^V1 6 Nursing- World.?An Acceptable Gift?The
Presentafi^ i?)1 Children? Workers Wanted?A Pleasant
caster?T.?? j ri Nurses?Whose Fault is It P?Wise Don-
San Rfinn *5;? Nurses for Wirksworth?English Nurses at
in, atled Nnr. ,.r?P0anB in India?A New Nurses' Home?Die-
to11? DeveW. The Hospital" Endowed Bed ?
??,e^evewtr',^eSoBnitar" E^dow^EnT" '". cxxiv
^Plfiss Piuieata toy Gymnastic/?. VII. cuxvii
Everybody's Opinion. ? American Methods ? American
Nurses?Presents and Presentations ? Patients* Meals?
" The HospitU "lEcdowed Bed cxxxviii
Cholera and Nurses cxxxix
Cholera Precautions?Appointments cxl
Minor Appointments?'Presentations - cxl
Por Reading to the Siok.?The Mnssaga .... oxi
Notes and Queries?"Wants and Workers .... Cxl

				

